# Late traumatic LASIK flap perforation and displacement by bird bite: A case report

**DOI:** 10.1097/MD.0000000000050404

**Published:** 2026-08-28

**Authors:** Shiuh-liang Hsu, Li-yi Chiu

**Affiliations:** a Department of Ophthalmology, Kaohsiung Medical University Chung-Ho Memorial Hospital, Kaohsiung, Taiwan; b Department of Ophthalmology, Kaohisung Medical University, Kaohsiung, Taiwan; c Graduate Institute of Clinical Medicine, Kaohsiung Medical University, Kaohsiung, Taiwan.

**Keywords:** flap dislocation, LASIK, YAG laser

## Abstract

**Rationale:**

Traumatic flap dislocation is a rare but vision-threatening complication following laser in situ keratomileusis (LASIK), which may occur even many years after surgery. We report a unique case of late traumatic LASIK flap perforation and displacement caused by a bird bite.

**Patient concerns:**

A 40-year-old woman presented with blurred vision and ocular irritation in her left eye 1 week after being bitten by her pet bird.

**Diagnoses:**

Slit-lamp examination and anterior segment optical coherence tomography revealed traumatic LASIK flap displacement with irregular flap edges, epithelial ingrowth, and a paracentral full-thickness flap perforation.

**Interventions:**

Urgent flap repositioning was performed on the same day of presentation. Recurrent epithelial ingrowth surrounding the perforation site was subsequently managed with lubrication using autologous serum eye drops and sequential Nd laser treatments.

**Outcomes:**

Visual acuity improved from 20/50 at presentation to 20/25 after treatment. No recurrent epithelial ingrowth was observed during long-term follow-up.

**Lessons:**

LASIK flaps remain vulnerable to traumatic displacement even many years after surgery. Prompt flap repositioning and sequential management of epithelial ingrowth can achieve favorable visual outcomes in complex traumatic cases involving flap perforation.

## 1. Introduction

Flap dislocation is one of the most severe complications following laser in situ keratomileusis (LASIK). Although uncommon, flap displacement may occur many years after surgery because the flap interface never completely regains normal corneal tensile strength. Previously reported causes included eye rubbing, blunt trauma, fingernail injuries, and sports-related trauma.^[1–4]^ However, traumatic flap perforation caused by a bird bite has not previously been reported.

We report a rare case of late traumatic LASIK flap displacement associated with full-thickness flap perforation caused by a bird bite. Sequential Nd laser treatments were required to manage recurrent epithelial ingrowth during long-term follow-up.

## 2. Case presentation

A 40-year-old woman presented to our outpatient clinic with blurred vision and ocular irritation in the left eye for 1 week after being bitten by her pet bird. She had undergone bilateral LASIK surgery 14 years previously. Her past ocular history was otherwise unremarkable, and no systemic autoimmune disease was reported.

Before presentation to our clinic, the patient had visited several local clinics and was initially diagnosed with corneal abrasion. However, her symptoms persisted despite topical treatment.

**Figure 1.**
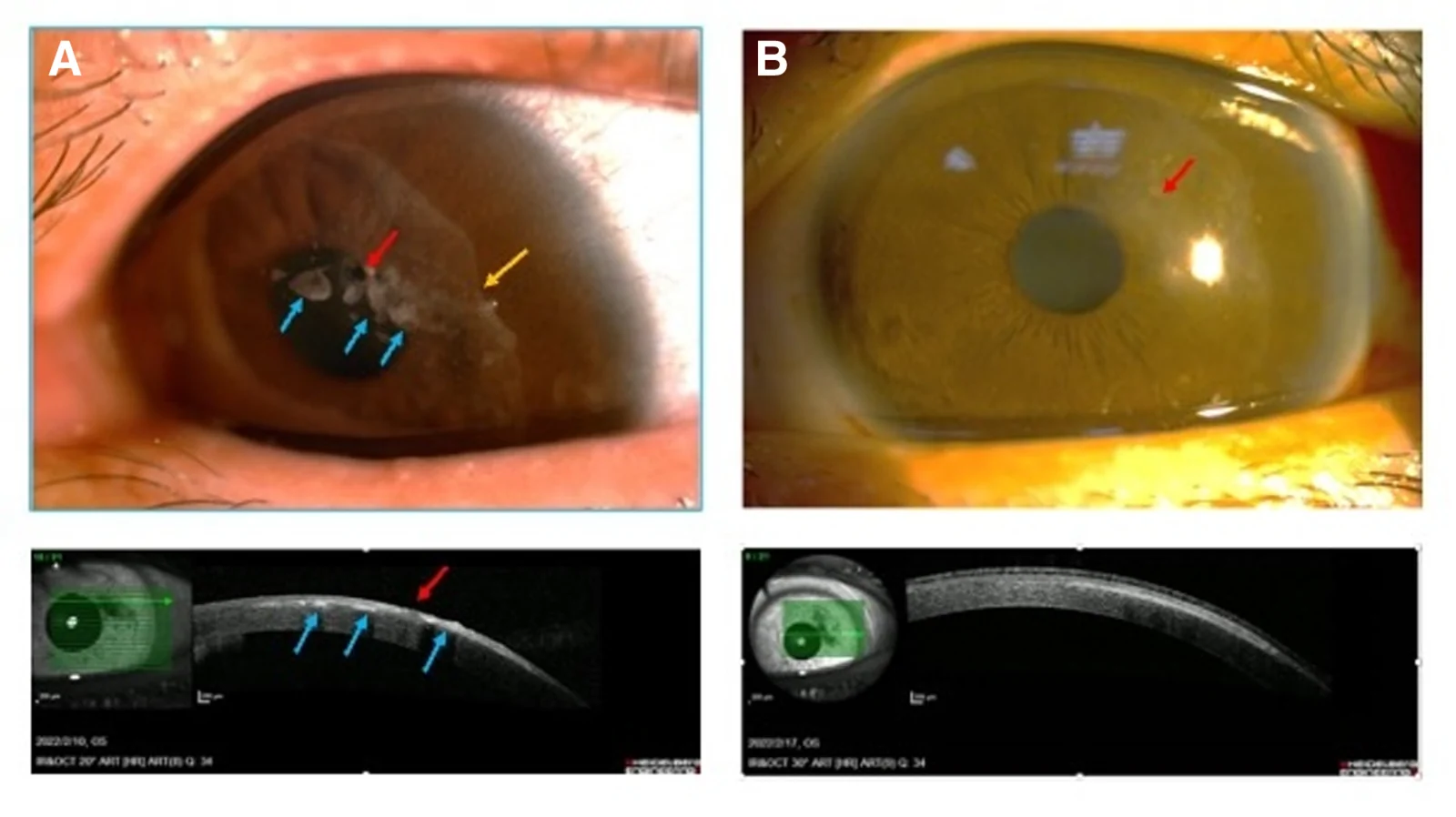
(A) Red arrow: full layer LASIK flap perforation; blue arrows: epithelium cells ingrowth; orange arrow: irregular flap edge. (B) Red arrow: full layer LASIK flap perforation. LASIK = laser in situ keratomileusis.

At presentation, corrected visual acuity in the affected eye was 20/50. Slit-lamp examination revealed traumatic LASIK flap displacement with irregular flap edges extending from 12 to 4 o’clock, associated with multiple epithelial ingrowth islands near the visual axis. A paracentral 1-mm full-thickness flap perforation was identified (Fig. 1A). Anterior segment optical coherence tomography demonstrated flap irregularity and hyperreflective interface lesions consistent with epithelial ingrowth (Fig. 1B).

Urgent flap repositioning was performed on the same day. The flap interface was carefully irrigated, and epithelial cells and debris were removed intraoperatively. A bandage contact lens was applied postoperatively. The patient received topical antibiotics, topical corticosteroids, preservative-free artificial tears, and autologous serum eye drops during follow-up.

**Figure 2.**
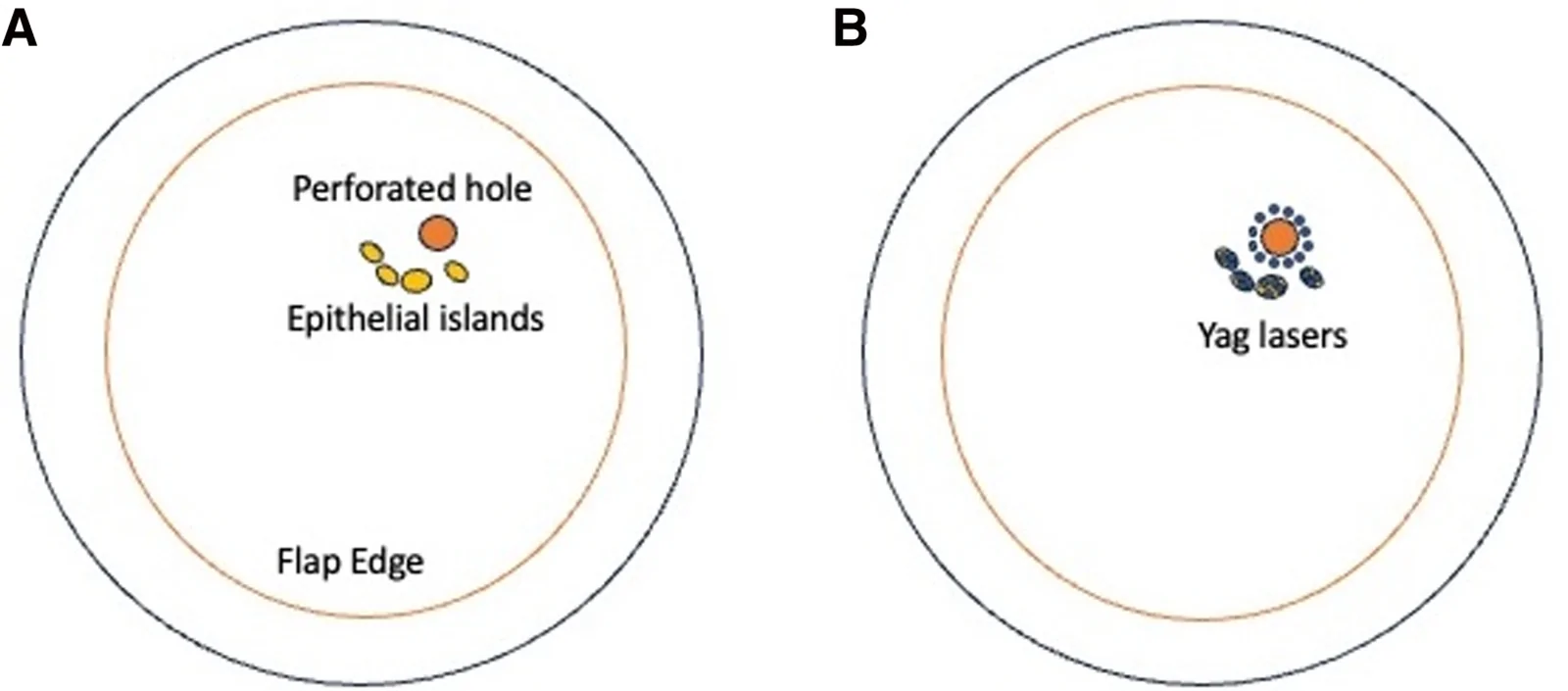
(A) Recurrent epithelial islands after surgery. (B) Nd-Yag lasers around hole and on the epithelial islands.

During postoperative follow-up, recurrent epithelial ingrowth developed around the perforation site noted 4 weeks after treatment (Fig. 2A). Sequential Nd:YAG laser treatments were therefore performed to ablate epithelial cells surrounding the perforation area and to inhibit further progression of epithelial ingrowth (Fig. 2B). The Nd:YAG laser energy settings ranged from 0.4 to 0.6 mJ, depending on the corneal response. Approximately 20 to 24 laser shots were delivered during each session. A total of 3 Nd:YAG laser treatments were performed at 4, 8, and 12 weeks after the initial surgery.

Visual acuity gradually improved to 20/25 after treatment. No recurrent epithelial ingrowth was observed during long-term follow-up.

## 3. Discussion

Traumatic LASIK flap displacement remains a clinically important complication even many years after surgery.^[1–4]^ Previous reports have demonstrated that late flap dislocation may occur after relatively minor trauma because the LASIK flap interface does not completely recover native stromal tensile strength.

In this case, the patient sustained a unique penetrating injury caused by a bird bite, resulting not only in flap displacement but also in full-thickness flap perforation. The perforation site increased the risk of recurrent epithelial ingrowth, which represented the major postoperative challenge.

Management of epithelial ingrowth following LASIK includes flap relifting with mechanical debridement, adjunctive ethanol or mitomycin-C treatment,^[5,6]^ suturing, fibrin glue, and Nd laser therapy. In our case, sequential Nd laser treatments were selected because the epithelial ingrowth was localized around the perforation site, and repeated flap lifting could increase flap instability. Nd laser treatment successfully controlled recurrent epithelial ingrowth without requiring additional flap surgery.

This case also highlights the importance of careful diagnostic examination in patients with persistent symptoms after ocular trauma, particularly in individuals with a remote history of LASIK surgery. The patient was initially diagnosed with corneal abrasion at outside clinics before flap displacement was recognized.

## 4. Conclusion

We report the first known case of traumatic LASIK flap perforation and displacement caused by a bird bite. Despite delayed presentation and recurrent epithelial ingrowth, prompt flap repositioning and sequential Nd laser treatments resulted in favorable long-term visual outcomes. Patients should be informed that LASIK flaps remain vulnerable to traumatic displacement even many years after surgery.

## Author contributions

**Data curation:** Shiuh-liang Hsu.

**Methodology:** Li-yi Chiu.

**Resources:** Li-yi Chiu.

**Writing – original draft:** Li-yi Chiu.

**Writing – review & editing:** Shiuh-liang Hsu, Li-yi Chiu.
